# Children’s Birthday Gatherings and SARS-CoV-2 Infection in Grandparents

**DOI:** 10.1001/jamanetworkopen.2026.23042

**Published:** 2026-07-13

**Authors:** Laura Espenhain, Laust Hvas Mortensen, Lasse Engbo Christiansen, Christian Holm Hansen, Steen Ethelberg

**Affiliations:** 1 Department of Infectious Disease Epidemiology and Prevention, Statens Serum Institut, Copenhagen, Denmark; 2 Department of Public Health, University of Copenhagen, Copenhagen, Denmark; 3 ROCKWOOL Foundation, Copenhagen, Denmark; 4 Statistics Denmark, Copenhagen, Denmark; 5 Department of Epidemiology Research, Statens Serum Institut, Copenhagen, Denmark

## Abstract

**Importance:**

During the COVID-19 pandemic, the major burden of morbidity and mortality was in older age groups. While within-household transmission is well documented, the wider role of social networks for transmission to the older population is less well understood.

**Objective:**

To estimate whether children’s birthdays were associated with grandparents’ SARS-CoV-2 infection risk.

**Design, Setting, and Participants:**

This nationwide, register-based cohort study included grandparents residing in Denmark with 1 to 5 grandchildren aged 0 to 15 years during the period between February 19, 2020, and February 28, 2022. The timing of birthdays of grandchildren was used as a natural experiment to examine whether birthday-related opportunity for family gatherings was associated with the risk of SARS-CoV-2 infection in grandparents. Family structures were mapped at the individual level. Data were analyzed from June 2024 to December 2026.

**Exposures:**

Birthdays of grandchildren turning 1 to 16 years. A birthday risk window, the period when positive test results could be ascribed to a celebration, was defined as the 7-day time window from 2 to 8 days after the grandchild’s birthday.

**Main Outcomes and Measures:**

The primary outcome was a positive result on a SARS-CoV-2 test (by polymerase chain reaction or antigen testing) recorded in the national surveillance system. Hazard ratios comparing the hazard of having positive test result for SARS-CoV-2 (outcome) in grandparents in birthday-windows vs grandparents who at the same moment were in nonbirthday periods, were estimated using Cox proportional hazards regression with birthdays as time-varying covariates.

**Results:**

Among 1 106 493 grandparents (median [IQR] age, 67 [60-73] years; 54% female), grandparents had 9.9% (95% CI, 7.9%-12.0%) higher hazard of SARS-CoV-2 infection during birthday risk windows. The hazard varied over time and by variant and was not seen during the period when Alpha dominated. The risk was greater for birthdays of preschool-aged grandchildren (adjusted hazard ratio, 1.15; 95% CI, 1.12-1.18) than during birthdays of school-aged grandchildren (adjusted hazard ratio, 1.07; 95% CI, 1.04-1.09).

**Conclusions and Relevance:**

This cohort study of grandparents found that grandchild birthdays, despite official advice not to gather during part of the pandemic, were associated with increased SARS-CoV-2 infection risk among grandparents during much of the pandemic, with magnitude of risk varying over time and by viral variant. These results emphasize the role of culturally important events in intergenerational transmission dynamics, supporting future targeted risk mitigation around small family events.

## Key Points

**Question:**

Are social gatherings, exemplified by children’s birthdays, associated with increased SARS-CoV-2 infection risk in older adults?

**Findings:**

In a cohort study of 1 106 493 grandparents with 961 294 grandchildren, the hazard of recorded SARS-CoV-2 infection was higher following grandchildren’s birthdays, a statistically significant association.

**Meaning:**

These findings suggest that small family events were a measurable transmission pathway to seniors, indicating that these socially important events were prioritized despite an ongoing pandemic; targeted risk-mitigation (eg, pre-event testing) might be a tool to reduce spill overs in future respiratory pandemics.

## Teaser

This cohort study uses Danish registry data to examine the association of grandchild birthdays, as a proxy for opportunity for social gathering, with SARS-CoV-2 infection in grandparents.

## Introduction

From its emergence in late 2019, COVID-19 quickly became a major concern to public health and the economy globally and drastically disrupted societal activities.^1,2,3^ Understanding the dynamics of the COVID-19 pandemic, including how the disease spread between population segments, remains essential for future pandemic response, yet these dynamics are challenging to measure directly.

The severity of COVID-19 increases sharply with age.^4,5,6,7,8,9^ Therefore, medical and nonmedical interventions initiated during the pandemic prioritized protecting older adults, and understanding transmission of disease into this group remains important. Older adults and other individuals at risk of severe COVID-19 disease were recommended to take a number of additional precautions to avoid COVID-19. In Denmark, such extra precautions included abstaining from taking part in social gatherings or, at a minimum, encouraging events to be carried out in a safe manner. However, carrying out gatherings in a safe manner could be challenging, as persons infected with SARS-CoV-2 may be infectious several days before onset of symptoms or may remain asymptomatic while infectious.^7,10,11,12^ In Denmark, authorities restricted gathering sizes in public spaces and recommended limits in private settings (initially ≤10 people, later ≤5) to reduce transmission.^13^ Nonetheless, cross-household intergenerational contact (eg, during social gatherings) may have been a pathway for introducing SARS-CoV-2 into groups at higher risk of severe COVID-19 outcomes. Quantifying the magnitude of this pathway is challenging because intergenerational mixing and contact are not directly observable in registers.

We addressed this challenge using a register-based natural experiment design. We hypothesized that children’s birthdays were occasions for family gatherings—even under policies that restricted behaviors—and thus created plausibly exogenous, short-term increases in intergenerational contact on or near the child’s birthday. We took advantage of Denmark’s extensive SARS-CoV-2 testing regimen during the pandemic, which ensured a high case ascertainment.^14,15^ Using Denmark’s administrative and health registers, we linked all grandparents to their grandchildren, test results, and demographic information. Treating each grandchild’s birthday as an exogenous marker of birthday-related opportunity for intergenerational contact during social gatherings, we estimate the associations of these social gatherings with grandparents’ SARS-CoV-2 infection risk.

## Methods

The register data used in this cohort study were collected under the legal obligation of the Statens Serum Institute as public authority set out in the Danish Health Act (Section 22). Individual informed consent is not required for register-based research projects that do not use biological specimens under Danish regulation. The project was submitted for ethical review and received internal approval from Statens Serum Institute. This study was reported in accordance with the Strengthening the Reporting of Observational Studies in Epidemiology (STROBE) reporting guideline.

### Study Design and Population

We carried out a nationwide register-based cohort study of grandparents who resided in Denmark during the period between February 19, 2020, and February 28, 2022. Eligible individuals were grandparents residing in Denmark and who had 1 to 5 grandchildren aged 0 to 15 years at 1 of the following fixed assessment dates: February 19, 2020; January 1, 2021; or January 1, 2022. Grandparents entered follow-up from the relevant assessment date at which they met the inclusion criteria. Our design exploits that birth dates are distributed independent of the pandemic context and seasonal and environmental factors. We used grandchildren’s birthdays as the observed exposure, treating them as an exogenous marker of a birthday-related opportunity for intergenerational contact. Grandparents were followed until the first of the following events: a positive SARS-CoV-2 test result, death, emigration, loss of eligibility, or the end of the study period on February 28, 2022. Loss of eligibility occurred if the grandparent no longer had 1 to 5 grandchildren aged 0 to 15 years according to the study definition.

### Exposure, Comparison, and Outcome

The primary exposure was the birthday of a grandchild, from the first to the 16th birthday. Guided by initial exploratory analyses, and to accommodate incubation time and variation in timing of the actual celebration, we defined the period when positive test results could be ascribed to a celebration as the 7-day time window from 2 to 8 days after the grandchild’s birthday. Since the February 29 did not exist in 2021 and 2022, birthdays on these 2 dates were moved to February 28. In 2 secondary analyses, we split the exposure into (1) birthday of a grandchild turning up to 6 years and birthday of a grandchild turning 7 to 16 years (age-model), and (2) birthday of a grandchild living less than 25 km away from the grandparent, living 25 to 149 km away from the grandparent, or living 150 km or farther away, defined from the centroids of the municipality of residence (distance-model).

The primary outcome was a positive SARS-CoV-2 test result (by polymerase chain reaction or antigen testing) recorded in the national surveillance system. As a secondary outcome, we examined death within 30 days of a positive test result; deaths were anchored to the date of the positive test result for temporal alignment with exposure (mors-model).

We present pooled estimates for the full study period and variant-specific estimates based on the dominant SARS-CoV-2 variant circulating in Denmark at the time: index (February 19, 2020, to January 4, 2021), Alpha (February 15, 2021, to June 21, 2021), Delta (June 28, 2021, to December 6, 2021), and Omicron (December 20, 2021, to February 28, 2022). As an exploratory analysis, we estimated hazard ratios (HRs) in sliding calendar-time windows of 4 weeks, moved in 1-day steps, to visualize temporal variation in the association between birthday-related exposure windows and SARS-CoV-2 infection risk over the course of the pandemic.

### Data Sources

The data used in this study are curated by Statistics Denmark and are based on the Danish Population registry and Danish COVID-19 surveillance, which includes test results of all SARS-CoV-2 polymerase chain reaction and antigen tests carried out in Denmark.^16,17^ Familial relationships (ie, grandparent-grandchild pairs), were identified via the population network build from the Danish Population registry.^18^ From the population registry, we additionally got information on registered addresses (municipalities), dates of death, and emigration or immigration.

### Statistical Analysis

We estimated the HRs comparing the hazard of having test result positive for SARS-CoV-2 (outcome) in grandparents in birthday windows vs grandparents who at the same moment were in nonbirthday periods, using Cox proportional hazards regression with birthdays as time-varying covariates. Statistical significance was indicated by a 2-sided *P* < .05. To tightly control for shared calendar-time factors, such as seasonal conditions, pandemic phase, and contemporaneous restrictions, calendar time served as the underlying time scale. To flexibly accommodate potential baseline risk differences by family size, we stratified the baseline hazard by number of grandchildren (1-5). In the secondary analyses, the age model additionally adjusted for whether the grandparent had grandchildren in the 0 to 6 years and/or 7 to 16 years age groups, as well as age groups (in 4 categories) of the grandparent and presented as adjusted HR (aHR); the distance-model was adjusted for the set of distance categories represented among the grandparent’s grandchildren; and the mors model was adjusted for age and sex of the grandparent. In secondary analyses, differences between the coefficients for the categories of age of the birthday child and distance between grandchild and grandparents were assessed using Wald tests of coefficient equality.

Although the registers used did not contain any missing information, antigen tests carried out at home is not a part of the Danish COVID-19 surveillance. No attempt to include these in the analyses was done.

#### Permutation Validation

We reassigned birthdays at random among grandchildren while preserving the overall data structure and reestimated HRs to gauge whether observed associations could arise from spurious correlations. We repeated the analyses 100 times and summarized the simulated HR point estimate distribution by means and 5th and 95th percentiles.

#### Risk-Window Variation

To assess robustness of the exposure period definition, we varied the start bound from 2 days before to 4 days after and the end bound from 4 to 14 days after the birthday. We visualized the results in a heatmaps of HR point estimates, with markers indicating combinations for which the lower bound of the 95% CI exceeded 1, across all risk-window start and end dates relative to the birth date and stratified by variant.

#### Subgroup and Association Modification

To test whether the HR of a positive test result for SARS-CoV-2 during a birthday-period varied by the sex or age of that grandparent, or the number of grandchildren, we included the variable in question in the Cox proportional hazard model as an interaction term. We considered the HR as varying significantly if the interaction term was significant or if the model fit was better than without, tested using analysis of variance log-likelihood ratio test.

Data preparation and analyses were conducted using Rstudio version 2025.05.1 and R version R-4.5.0 (R Project for Statistical Computing). Among others, the data.table package (version 1.17.4) was used to split data into relevant intervals in preparation for Cox regression, and coxph and cox.zph from the survival package (version 3.8-3) were used to carry out the Cox proportional hazards regression and test for proportional hazards. We used ggplot2 for visualizations. Data were analyzed from June 1, 2024, to December 1, 2025.

## Results

**Figure 1.**
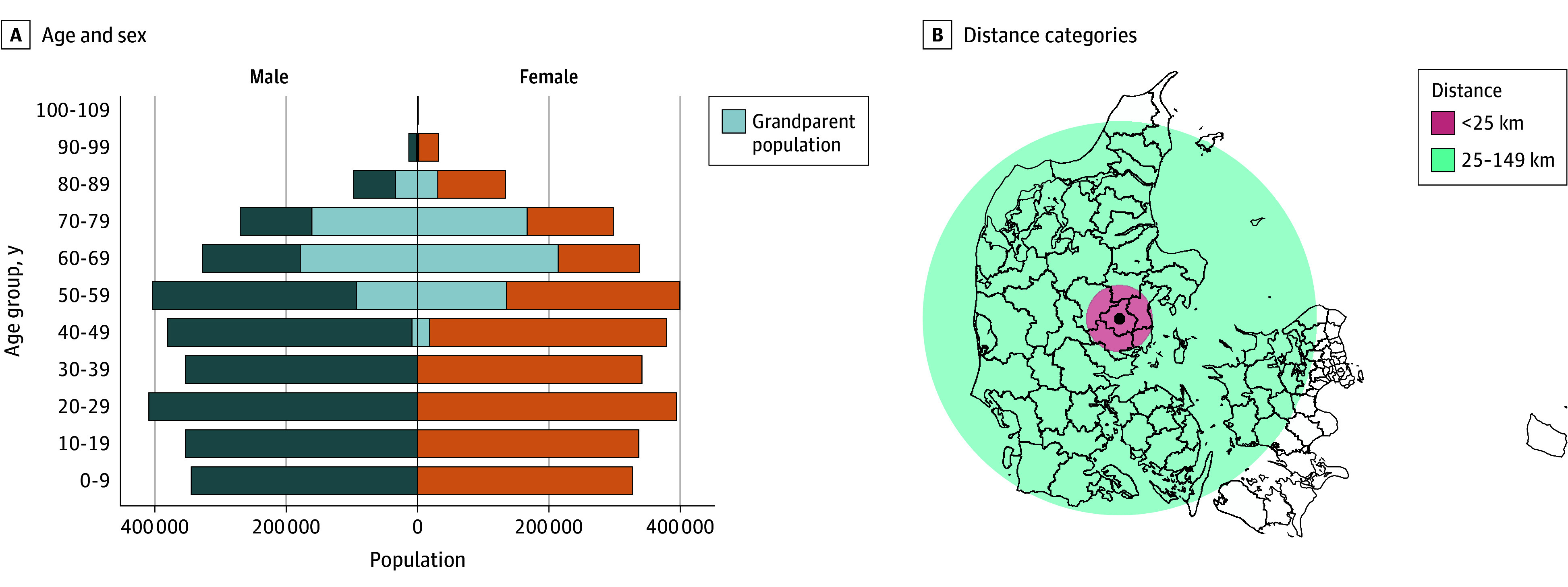
Pyramid of the Characteristics of the Study Population and Map of Denmark A, As of February 19, 2020. B, Defined from the centroids of the municipality of residence.

**Figure 2.**
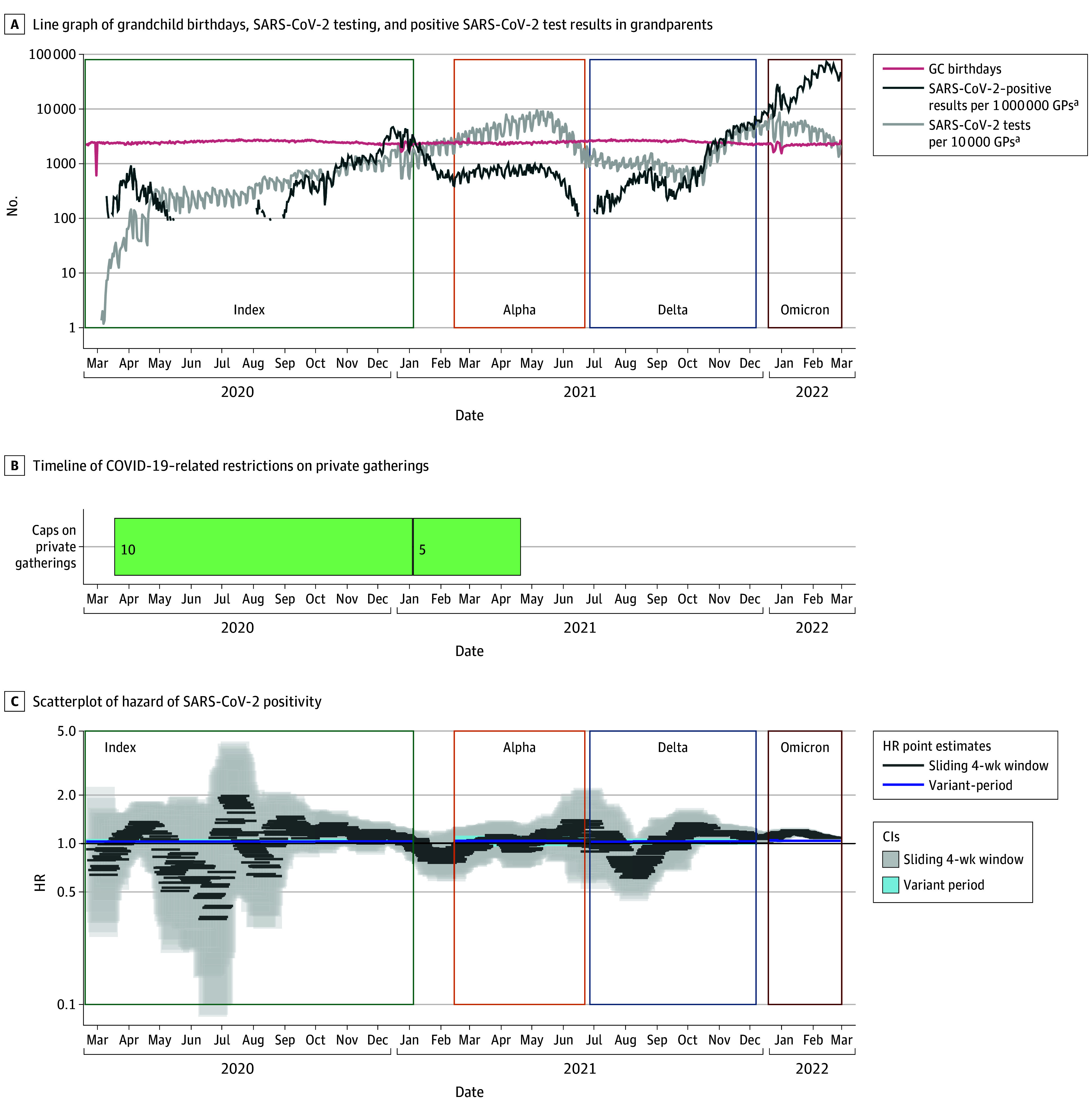
Overview of the Study Period GC indicates grandchild; GP, grandparent; HR, hazard ratio. ^a^Minimum absolute number shown is 10.

In this nationwide cohort we followed 1 106 493 grandparents for 2 032 596 person-years during the period between February 19, 2020, and February 28, 2022 (eFigure in Supplement 1). The median (IQR) age of the cohort was 67 (60-73) years, 54.2% were female, and 57.8% had 1 or 2 grandchildren. These grandparents were linked to 961 294 unique grandchildren. The age and sex distribution is visualized in Figure 1A. A full descriptive characteristic of the study population is available in the Table. Grandchild birthdays were broadly distributed through the year, with slightly more birthdates in summer and late September and marked dips on February 29 and December 24 to 26. Birthday windows (days 2 to 8 following a birthday) accounted for 3.5% to 3.9% of total person-time (Table). During the study period, 27.5% of grandparents had a test result positive for SARS-CoV-2 across 4 epidemic waves, mostly during the Omicron wave (Figure 2). The test activity varied over the study period and peaked during the Alpha variant period and the beginning of the Omicron variant period (Figure 2).

**Table.** Descriptive Characteristics of the Study Population Overall and by Variant Period

Variable	Period, No.									
	Entire study period^a^		Index^b^		Alpha^c^		Delta^d^		Omicron^e^	
	PY per 10 000 population	Positives per 100 PY	PY per 10 000 population	Positives per 100 PY	PY per 10 000 population	Positives per 100 PY	PY per 10 000 population	Positives per 100 PY	PY per 10 000 population	Positives per 100 PY
Total (N = 1 106 493)	203	15	90	3.2	35	2.4	44	5.2	17	139
Birthday risk window	8	16	3	3.2	1.3	2.4	1.7	5.5	0.6	159
Sex										
Female	110	16	49	3.3	19	2.3	24	5.2	8.9	145
Male	93	14	41	3.1	16	2.4	20	5.2	7.6	130
Age, y										
<60	47	21	22	4.1	8.2	4.5	10	7.0	3.2	225
60-66	52	17	23	3.2	9.0	2.6	11	5.3	4.2	162
67-74	64	12	28	2.5	11	1.4	14	4.7	5.3	107
≥75	41	11	17	3.3	6.9	0.9	9.4	3.9	3.8	83
GC, No.										
1	60	16	27	4.0	10	2.5	13	5.1	5.0	137
2	64	14	28	2.4	11	2.2	14	5.0	5.3	134
3	37	15	17	2.4	6.4	2.3	8.0	5.3	3.0	139
4	27	15	12	2.7	4.7	2.4	6.0	5.6	2.2	147
5	14	17	6.3	6.3	2.5	2.6	3.1	5.5	1.1	150
Closest GC, km										
24	137	16	61	3.5	23	2.6	30	5.5	11	145
25-149	54	14	24	2.7	9.3	1.9	12	4.4	4.5	127
≥150	12	13	5.4	2.6	2.1	1.7	2.7	4.5	1.0	121
Has school- and non–school-aged GC	58	16	26	3.3	10	2.5	13	5.5	4.7	151
Has only school-aged GC	63	18	28	3.4	11	3.5	14	6.2	4.5	188
Has only non–school-aged GC	83	12	37	3.0	14	1.3	18	4.2	7.3	99

Abbreviations: GC, grandchild; PY, person-year (accumulated by 2 genders, 4 age groups, number of grandchildren, 3 distance categories, and 3 categories of GC).

^a^ February 19, 2020, to February 28, 2022.

^b^ February 19, 2020, to January 4, 2021.

^c^ February 15, 2021, to June 21, 2021.

^d^ June 28, 2021, to December 6, 2021.

^e^ December 20, 2021, to February 28, 2022.

### Association Between Birthdays and SARS-CoV-2 Infection

**Figure 3.**
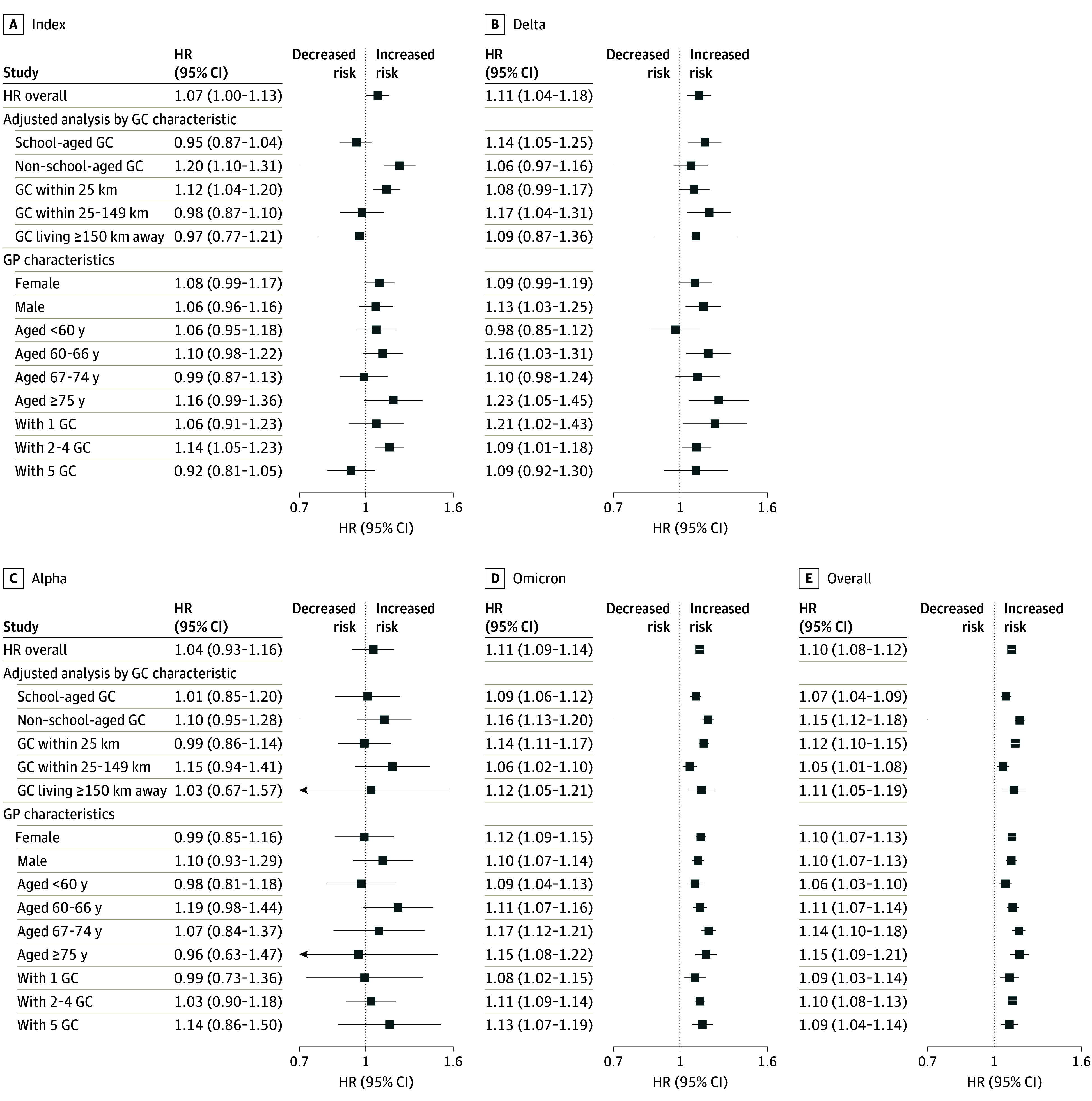
Forest Plots of Hazard of SARS-CoV-2 Infection in Grandparents (GPs) Associated With Birthdays in Their Grandchildren (GC) Results of Cox regression analyses adjusted for number of grandchildren and shown by the 4 time periods dominated by different viral variant and the overall study period. Adjusted hazard ratios (HRs) are presented for birthday periods by age of the birthday GC, adjusted for number of GC and whether the grandparent had a grandchild in the age category and for birthday periods by geographical proximity of the birthday GC, adjusted for number of GC and whether the GP had a GC in the distance category. Analyses by GP characteristics are unadjusted.

Across the full study period, a grandparent in a birthday risk period had a 9.9% (95% CI, 7.9%-12.0%) higher hazard of having a test result positive for SARS-CoV-2 than a grandparent who at the same time was in a nonbirthday period (Figure 3; eTable 1 in Supplement 1). In secondary analyses, the risk was greater for birthdays of younger children of preschool age (aHR, 1.15; 95% CI, 1.12-1.18) than during birthdays of school-aged grandchildren (turning 7-16 years: aHR, 1.07; 95% CI, 1.04-1.09) (Figure 3; eTable 2 in Supplement 1), and this difference was statistically significant (Wald test: *P* < .001). Risks were also increased in subgroup analyses focusing on grandparents older than retirement age (≥67 years) (non–school aged-grandchildren: aHR, 1.22 [95% CI, 1.15-1.29]; school-aged grandchildren: aHR, 1.12 [95% CI, 1.08-1.15]; Wald test: *P* = .005).

When stratifying by the dominant variant, hazards were significant for all periods except the Alpha period (index: aHR, 1.07 [95% CI, 1.00-1.13]; Alpha: aHR, 1.04 [95% CI, 0.93-1.16]; Delta: aHR, 1.11 [95% CI, 1.04-1.18]; Omicron: aHR, 1.11 [95% CI, 1.09-1.14]) (Figure 3; eTable 1 in Supplement 1). The association of birthdays with COVID-19 risk did not vary materially by sex or number of grandchildren linked to the grandparent (Figure 3; eTable 4 in Supplement 1). During the index period, there was no increased hazard of positive test results following a birthday in the age 67 years or older groups, whereas during the Omicron variant period, the hazard was higher in the age 67 to 74 years group.

During the index and the Omicron variant periods, in secondary analyses, the hazard of having a test result positive for SARS-CoV-2 following a birthday in a grandchild turning 1 to 6 years was higher than for that following a birthday in a grandchild aged 7 to 16 years (index period: aHR, 1.20 [95% CI, 1.10-1.31] vs aHR, 0.95 [95% CI, 0.87-1.04]; Wald test: *P* < .001; Omicron period: aHR, 1.16 [95% CI, 1.13-1.20] vs aHR, 1.09 [95% CI, 1.06-1.12]; Wald test: *P* = .002). We observed the opposite during the Delta variant period, when there was only a significantly higher hazard of testing positive during birthday windows of school-aged children (aHR, 1.06 [95% CI, 0.97-1.16] vs aHR, 1.14 [95% CI, 1.05-1.25]; Wald test: *P* = .26) (Figure 3; eTable 2 in Supplement 1). The association of birthdays with SARS-CoV-2 positivity varied with geographical proximity between grandparent and birthday grandchild (Figure 3; eTable 3 in Supplement 1).

**Figure 4.**
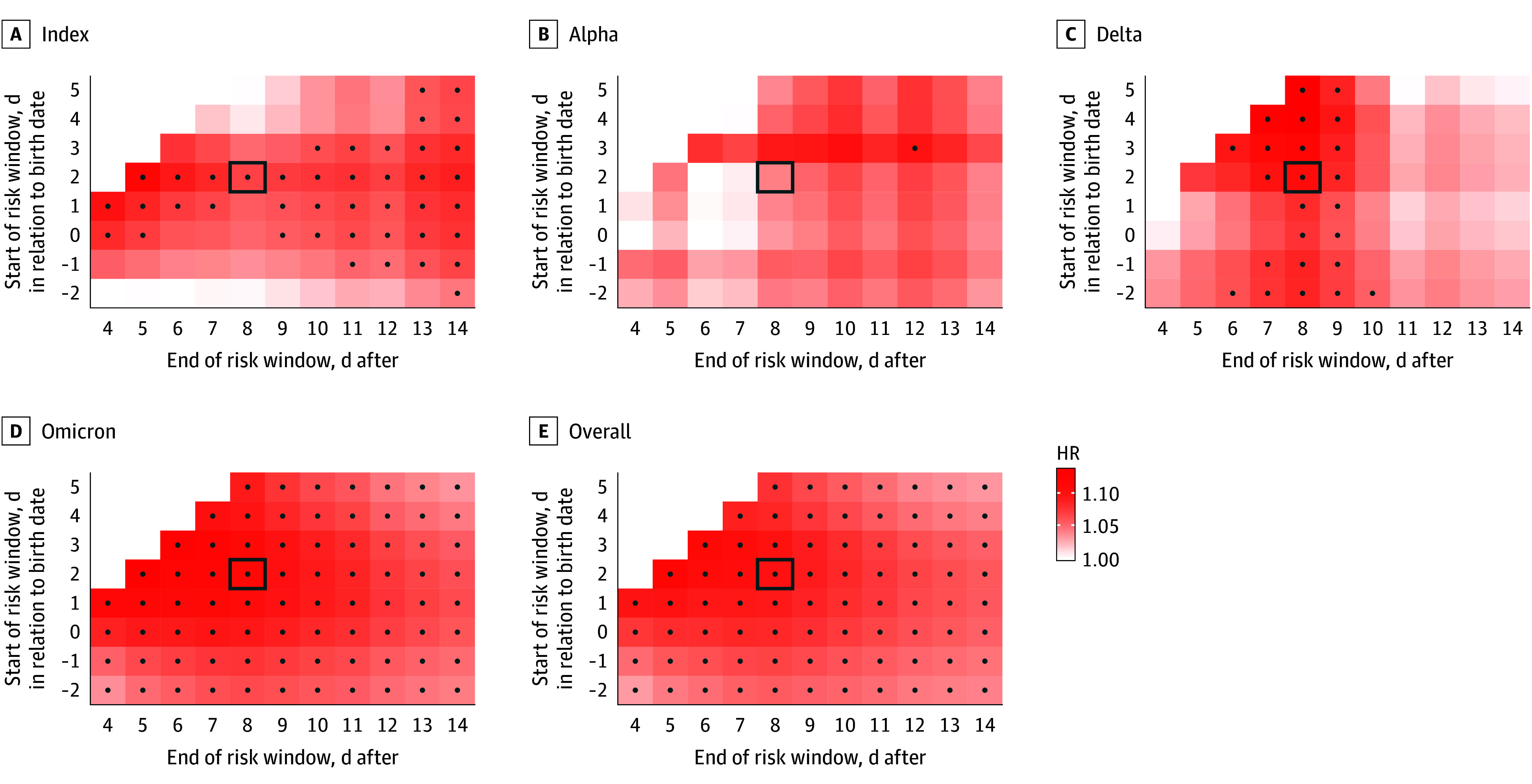
Heat Maps of Hazard of SARS-CoV-2 Infection by Varying Starts and Ends of Birthday Exposure Windows Dots indicate that the lower bound of the 95% CI is greater than 1; squares indicated the exposure period in the main analyses. HR indicates hazard ratio.

Analyses varying the start, end, and duration of the time window around the birthdays indicated that the association was concentrated in short postbirthday periods, with the strongest signals occurring in the days immediately after the birthday (Figure 4). During the index and Omicron variant periods, the clearest signals were seen in windows starting 2 to 4 days after the birthday (Figure 4). During the Delta variant period, the strongest signals were seen in windows spanning approximately 4 to 8 days after the birthday (Figure 4).

In the exploratory sliding-window analysis, the estimated association varied over calendar time (Figure 2C). Estimates fluctuated early in the pandemic, when case numbers and test activity were lower and precision limited. The point estimates rose above 1 from the end of August 2020 and remained near or above 1 until mid-December 2020, after which a decrease in HRs was present until mid-January 2021. During the first part of the Alpha variant period, the point estimates fluctuated near 1 and increased toward the end and until Delta took over.

### Secondary Outcome: Death Within 30 Days of a Positive Test Result

We assessed death within 30 days of a positive SARS-CoV-2 test as a secondary outcome. Overall, we observed no association between birthday windows and 30-day mortality after a positive test result, except during the Alpha period, when no overall association was seen for infection (overall: aHR, 1.04 [95% CI, 0.98-1.10]; index: aHR, 0.97 [95% CI, 0.88-1.06]; Alpha: aHR, 1.18 [95% CI, 1.03-1.35]; Delta: aHR, 1.02 [95% CI,0.90-1.15]; Omicron: aHR, 1.14 [95% CI, 0.93-1.38]). No overall association between birthday windows and 30-day mortality after a positive test result was seen among grandparents aged 67 years or older (overall: aHR, 1.04 [95% CI, 0.97-1.11]).

### Validation Analysis

In the permutation analyses using randomly shuffled birthdays (100 simulations), there was no association with infection in the overall study period (HR, 1.00; 95% CI, 0.98-1.02). We also did not find any significant results in subanalyses for the index (HR, 1.00; 95% CI, 0.95-1.06), Alpha (HR, 0.99; 95% CI, 0.90-1.10), Delta (HR, 1.00; 95% CI, 0.94-1.06), or Omicron (HR, 1.00; 95% CI, 0.98-1.02) periods.

## Discussion

In this national cohort study, we used grandchildren’s birthdays as an exogenous marker of opportunity for intergenerational contact during gatherings to study whether these social gatherings were associated with a measurable risk of SARS-CoV-2 infection for older adults. We found that grandparents had an increased risk of having a positive test result for SARS-CoV-2 in the 7-day period following the birthdays of their grandchildren. The association varied over the course of the pandemic period and was not observed during the period when the Alpha variant dominated. The association was generally stronger for birthdays of younger, non–school-aged grandchildren, except during the period when the Delta variant dominated.

During the pandemic, a large variety of nonpharmaceutical interventions, including bans of larger gatherings, were in place.^13^ In Denmark, the population was informed about the risks through mass media and there was a clear public narrative of the need to protect older adults and individuals at risk of severe COVID-19 outcomes.^19,20^ In the first year of the pandemic, and possibly longer, the recommendation of cancelling private gatherings with more than 10 guests was widely accepted.^21,22^ Nevertheless, when looking at the first year of the pandemic, we did find that grandparents were more likely to have a positive test result when their grandchildren had birthdays, suggesting that social gatherings remained epidemiologically relevant. The importance of taking social science factors into consideration when doing epidemic control has been shown over multiple epidemics, eg, Ebola and HIV/AIDS.^23,24,25,26,27^ Even in a generally well-informed population with high trust in the authorities, like in Denmark,^28^ cultural and social factors are important drivers of behavior. In the first months of 2021, the limit on how many people were allowed to gather was lowered to 5 persons because the more infectious Alpha variant appeared and options for fast and repeated pre-event testing and, importantly, vaccination were introduced, which likely explain why we did not see an elevated infection risk around grandchildren’s birthdays in this period.

Our approach builds on prior work by Whaley et al,^29^ who used birthdays as a natural experiment to study COVID-19 diagnoses in US households during the first part of the pandemic. As in that study, we found that birthday-related periods were associated with increased SARS-CoV-2 risk. However, the 2 studies address different epidemiological questions. Whereas Whaley et al^29^ examined household diagnoses following birthdays within the household, we extended the approach to a nationwide individual-level study and included intergenerational links. Key strengths of our study compared with the study by Whaley et al^29^ include full population coverage, family linkage at the level of individuals, nationwide test data from a highly tested population, and the use of a Cox model with calendar time as the underlying timescale, allowing comparison of exposed and unexposed grandparents on the same dates. Thus, our findings complement the US evidence and suggest that birthday-related family contact remained epidemiologically relevant across 2 different settings, although the magnitude and expression of the association may differ between settings.

To our knowledge, no prior study has estimated birthday-associated intergenerational contact in registries. Related evidence comes from survey-based analyses from a multicountry study covering 27 European countries and Israel, reporting an association of grandparental childcare with higher risk of COVID-19.^30^ Other studies have looked at intergenerational coresidence and the risk or severity of COVID-19 in grandparents with mixed results.^30,31,32,33^ Others have reported lower risk of severe COVID-19 in adults with exposure to children compared with adults without.^34^ In Denmark, few grandparents (2%) cohabit with their grandchildren,^35^ and in social contacts and mixing studies, people 60 years and older reported fewer contacts than most other age groups.^36^

In line with the broader literature,^37,38,39,40,41,42^ we detected higher hazards during the Delta and Omicron periods compared with the index period. However, intergenerational contact during social gatherings and the risk of infection associated with it will, apart from transmissibility, depend on individual factors, such as risk aversion behavior and time period (eg, level of restrictions, availability of vaccines or tests, options for being outdoors). We observed no association during the Alpha variant period. Several contextual factors may have contributed to this observation. In the first months of 2021, the limit on how many could gather in public places was lowered from 10 to 5 individuals because of concern about the Alpha variant.^13^ This may have reduced or postponed birthday-related family gatherings and could help explain why we did not observe an association during this period. In addition, vaccination of older adults had just begun, and some grandparents may have delayed participation in social events until after vaccination. Antigen testing was also scaled up during this period, and pre-event testing was widely encouraged or required in many settings^13^ which may have reduced infection risk when gatherings occurred.^43^ We observed an overall association during the Delta and Omicron periods. These were periods with fewer unknowns: a vaccinated population, 2 years of experience with COVID-19, fewer restrictions in place, and highly infectious viruses.^13^

### Strengths and Limitations

This study has both strengths and limitations. A major strength is the nationwide linkage of grandparents and grandchildren at the individual level in a setting with extensive SARS-CoV-2 testing. In addition, the permutation analysis with randomly reassigned birthdays did not show similar associations, which supports the validity of the main findings. The timing analysis supports the interpretation that the observed association was short-lived and temporally aligned with the birthday; however, the subgroup and timing analyses should be interpreted as supportive and exploratory, as they were intended to contextualize the primary findings rather than to establish separate primary associations.

Our findings are also subject to additional limitations. The Omicron variant period accounted for three-fourths of all SARS-CoV-2 infections during the study period and may have driven the overall result; however, we took this into account by performing the variant period–specific analyses. Our results are indirect in the sense that the observed exposure was the grandchild’s birth date rather than a directly observed birthday gathering. Therefore, the design should be interpreted as analogous to an intention-to-treat framework: the birthday is the observed exogenous trigger, whereas the actual gathering is an unobserved behavioral response. To the extent that some birthdays did not lead to gatherings—for example, because family members did not prioritize or risk gathering, lived far from their grandchildren, or were not invited—would have resulted in nondifferential misclassification of the exposure and likely have attenuated the estimated association relative to the effect of actual birthday-related gatherings. Whether and how birthdays were celebrated likely varied with season, pandemic phase, public health restrictions, and individual risk perception. Even within the group of people who did actually meet, adherence to recommendation for social distancing and opportunities for celebrating outside or in big spaces may have affected the individual risk.^44^ The estimated birthday-related association varied over time, but did not appear to track background SARS-CoV-2 incidence in a simple one-to-one manner. This is important to interpret in light of the Cox model, which estimates relative associations on the hazard scale. The use of calendar time as the underlying timescale in the Cox model ensured close control for shared time-varying background factors, as grandparents in a birthday risk window on a given date were compared with grandparents not in a birthday risk window on that same date. We were also not able to identify the exact source of transmission, only that infection risk was elevated during the period following the birthday. Therefore, infection may have been introduced by other birthday guests or through related contacts rather than by the grandchild or the celebration itself.

Furthermore, generalizability may be limited, as Denmark may differ from other settings in several potentially important respects, such as easy access to free testing, public health recommendations, public trust, and behavioral responses during the pandemic. Although our findings were broadly consistent with those of Whaley et al,^29^ the magnitude and expression of birthday-related transmission risk may therefore differ across settings.

## Conclusions

In this cohort study of grandparents, periods following grandchildren’s birthdays were associated with increased SARS-CoV-2 infection risk during much of the pandemic, despite epidemic control restrictions and free and widely available testing. The magnitude varied over time and by viral variant. These findings reemphasize how social events may have been perceived as more important than risk-limiting behaviors and support future targeted risk mitigation around small family events in future respiratory epidemics.

## Notes

Supplement 1. eTable 1. Hazard ratio of birthday of a grandchild eTable 2. Hazard ratio of birthday of a grandchild by age of the grandchild eTable 3. Hazard ratio of birthday of a grandchild by geographical distance between the grandparent and the grandchild eTable 4. Overall and by variant-period: HR and 95%CI of cox proportional hazard regression of the effect of a grandchild’s birthday eFigure. Flow diagram

Supplement 2. Data Sharing Statement

## References

[1] World Health Organization. WHO Director-General’s statement on IHR Emergency Committee on Novel Coronavirus (2019-nCoV). Accessed September 15, 2025. https://www.who.int/news-room/speeches/item/who-director-general-s-statement-on-ihr-emergency-committee-on-novel-coronavirus-(2019-ncov)

[2] Bollyky TJ, Castro E, Aravkin AY, . Assessing COVID-19 pandemic policies and behaviours and their economic and educational trade-offs across US states from Jan 1, 2020, to July 31, 2022: an observational analysis. Lancet. 2023;401(10385):1341-1360. doi:10.1016/S0140-6736(23)00461-0 36966780 PMC10036128

[3] Simonsen L, Pedersen RK, Andreasen V, Krause TG, Petersen E. A disease suppression strategy in action: The impact of non-pharmaceutical interventions in the COVID-19 pandemic in Denmark. Int J Infect Dis. 2025;160:108039. doi:10.1016/j.ijid.2025.108039 40907738

[4] Wolff D, Nee S, Hickey NS, Marschollek M. Risk factors for COVID-19 severity and fatality: a structured literature review. Infection. 2021;49(1):15-28. doi:10.1007/s15010-020-01509-1 32860214 PMC7453858

[5] Gao YD, Ding M, Dong X, . Risk factors for severe and critically ill COVID-19 patients: a review. Allergy. 2021;76(2):428-455. doi:10.1111/all.14657 33185910

[6] Zhou F, Yu T, Du R, . Clinical course and risk factors for mortality of adult inpatients with COVID-19 in Wuhan, China: a retrospective cohort study. Lancet. 2020;395(10229):1054-1062. doi:10.1016/S0140-6736(20)30566-3 32171076 PMC7270627

[7] Wu Z, McGoogan JM. Characteristics of and important lessons from the coronavirus disease 2019 (COVID-19) outbreak in China: summary of a report of 72 314 cases from the Chinese Center for Disease Control and Prevention. JAMA. 2020;323(13):1239-1242. doi:10.1001/jama.2020.2648 32091533

[8] Guan WJ, Ni ZY, Hu Y, ; China Medical Treatment Expert Group for COVID-19. Clinical characteristics of coronavirus disease 2019 in China. N Engl J Med. 2020;382(18):1708-1720. doi:10.1056/NEJMoa2002032 32109013 PMC7092819

[9] Wu C, Chen X, Cai Y, . Risk factors associated with acute respiratory distress syndrome and death in patients with coronavirus disease 2019 pneumonia in Wuhan, China. JAMA Intern Med. 2020;180(7):934-943. doi:10.1001/jamainternmed.2020.0994 32167524 PMC7070509

[10] Qiu X, Nergiz AI, Maraolo AE, Bogoch II, Low N, Cevik M. The role of asymptomatic and pre-symptomatic infection in SARS-CoV-2 transmission—a living systematic review. Clin Microbiol Infect. 2021;27(4):511-519. doi:10.1016/j.cmi.2021.01.011 33484843 PMC7825872

[11] Abdul-Rahman T, Nazir A, Khater B, . Increased rhinovirus/enterovirus infections including Ev-D68 in the United States, a challenge for healthcare providers amidst influenza virus infection and the COVID-19 pandemic. Postgrad Med J. 2023;99(1171):372-374. doi:10.1093/postmj/qgad016 36841228

[12] Bai Y, Yao L, Wei T, . Presumed asymptomatic carrier transmission of COVID-19. JAMA. 2020;323(14):1406-1407. doi:10.1001/jama.2020.2565 32083643 PMC7042844

[13] Munch PK, Espenhain L, Hansen CH, Krause TG, Ethelberg S. Case-control study of activities associated with SARS-CoV-2 infection in an adult unvaccinated population and overview of societal COVID-19 epidemic counter measures in Denmark. PLoS One. 2022;17(11):e0268849. doi:10.1371/journal.pone.0268849 36383627 PMC9668151

[14] Gram MA, Steenhard N, Cohen AS, . Patterns of testing in the extensive Danish national SARS-CoV-2 test set-up. PLoS One. 2023;18(7):e0281972. doi:10.1371/journal.pone.0281972 37490451 PMC10368237

[15] Krogsgaard LW, Espenhain L, Tribler S, . Seroprevalence of SARS-CoV-2 antibodies in Denmark: results of two nationwide population-based surveys, February and May 2021. Infect Drug Resist. 2023;16:301-312. doi:10.2147/IDR.S383491 36683911 PMC9851711

[16] Schmidt M, Pedersen L, Sørensen HT. The Danish Civil Registration System as a tool in epidemiology. Eur J Epidemiol. 2014;29(8):541-549. doi:10.1007/s10654-014-9930-3 24965263

[17] Witteveen-Freidl G, Lauenborg Møller K, Voldstedlund M, Gubbels S; Statens Serum Institut COVID-19 Automated Surveillance Group. Data for action—description of the automated COVID-19 surveillance system in Denmark and lessons learnt, January 2020 to June 2024. Epidemiol Infect. 2025;153:e58. doi:10.1017/S0950268825000263 40082077 PMC12001143

[18] Cremers J, Kohler B, Maier BF, . Unveiling the social fabric through a temporal, nation-scale social network and its characteristics. Sci Rep. 2025;15(1):18383. doi:10.1038/s41598-025-98072-2 40419631 PMC12106742

[19] Sundheds-Ældreministeriet. Aftale om initiativer for svækkede ældre i forbindelse med COVID-19. May 1, 2020. Accessed September 15, 2025. https://regeringen.dk/media/r30edtta/aftale-om-initiativer-for-svaekkede-aeldre-i-forbindelse-med-covid.pdf

[20] Sundhedsstyrelsen. Hvad kan ældre og personer med kronisk sygdom gøre for at beskytte sig mod coronavirus/COVID-19? March 12, 2020. Accessed September 15, 2025. https://www.sst.dk/da/nyheder/2020/Hvad-kan-aeldre-og-personer-med-kronisk-sygdom-goere-for-at-beskytte-sig-mod-coronavirus-COVID-19

[21] Petersen MB, Lindholt MF, Jørgensen F. Håndteringen af Coronaepidemien og Borgernes Adfærd, Tillid og Trivsel. Accessed September 15, 2025. https://fm.dk/media/vupjoa4b/3-baggrundspapir-ha-ndteringen-af-coronaepidemien-og-borgernes-adfaerd-tillid-og-trivsel_a.pdf

[22] Petersen MB, Bor A. Hvordan danner borgerne holdninger til restriktioner mod COVID-19? Accessed September 2, 2025. https://raw.githubusercontent.com/mariefly/HOPE/master/Opbakning_til_restriktioner_20210209.pdf

[23] Janes CR, Corbett KK, Jones JH, Trostle J. Emerging infectious diseases: the role of social sciences. Lancet. 2012;380(9857):1884-1886. doi:10.1016/S0140-6736(12)61725-5 23200487

[24] Anoko JN, Barry BR, Boiro H, . Community engagement for successful COVID-19 pandemic response: 10 lessons from Ebola outbreak responses in Africa. BMJ Glob Health. 2020;4(suppl 7):e003121. doi:10.1136/bmjgh-2020-003121 32816819 PMC7445346

[25] Williams HA, Jones CO. A critical review of behavioral issues related to malaria control in sub-Saharan Africa: what contributions have social scientists made? Soc Sci Med. 2004;59(3):501-523. doi:10.1016/j.socscimed.2003.11.010 15144761

[26] Bardosh KL, de Vries DH, Abramowitz S, . Integrating the social sciences in epidemic preparedness and response: a strategic framework to strengthen capacities and improve Global Health security. Global Health. 2020;16(1):120. doi:10.1186/s12992-020-00652-6 33380341 PMC7772799

[27] Gobat N, Carter S, Kutalek R, Rashid SF, Lees S, Anoko JN. Social science evidence for outbreak and pandemic response: rapid research and analytics for public health emergencies. In: Sorenson RA, ed. Principles and Practice of Emergency Research Response. Springer International Publishing; 2024:693-715. doi:10.1007/978-3-031-48408-7_39

[28] Lindholt MF, Jørgensen F, Bor A, Petersen MB. Public acceptance of COVID-19 vaccines: cross-national evidence on levels and individual-level predictors using observational data. BMJ Open. 2021;11(6):e048172. doi:10.1136/bmjopen-2020-048172 34130963 PMC8210695

[29] Whaley CM, Cantor J, Pera M, Jena AB. Assessing the association between social gatherings and COVID-19 risk using birthdays. JAMA Intern Med. 2021;181(8):1090-1099. doi:10.1001/jamainternmed.2021.2915 34152363 PMC8218234

[30] Uccheddu D, Rizzi EL. Intergenerational ties and COVID-19 contagion: a study on European adults aged 50 years and older using SHARE Data. J Gerontol B Psychol Sci Soc Sci. 2023;78(4):749-763. doi:10.1093/geronb/gbac196 36541727 PMC10439483

[31] Arpino B, Bordone V, Pasqualini M. No clear association emerges between intergenerational relationships and COVID-19 fatality rates from macro-level analyses. Proc Natl Acad Sci U S A. 2020;117(32):19116-19121. doi:10.1073/pnas.2008581117 32699150 PMC7431085

[32] Andrade FCD, Quashie NT, Schwartzman LF. Coresidence increases the risk of testing positive for COVID-19 among older Brazilians. BMC Geriatr. 2022;22(1):105. doi:10.1186/s12877-022-02800-6 35123395 PMC8817777

[33] Brandén M, Aradhya S, Kolk M, . Residential context and COVID-19 mortality among adults aged 70 years and older in Stockholm: a population-based, observational study using individual-level data. Lancet Healthy Longev. 2020;1(2):e80-e88. doi:10.1016/S2666-7568(20)30016-7 33521770 PMC7832817

[34] Solomon MD, Escobar GJ, Lu Y, . Risk of severe COVID-19 infection among adults with prior exposure to children. Proc Natl Acad Sci U S A. 2022;119(33):e2204141119. doi:10.1073/pnas.2204141119 35895714 PMC9388132

[35] Damm EA. Flere familier bor sammen på tværs af generationer. August 3, 2019. Accessed September 19, 2025. https://www.ae.dk/files/dokumenter/analyse/ae_flere-familier-bor-sammen-paa-tvaers-af-generationer.pdf

[36] Mossong J, Hens N, Jit M, . Social contacts and mixing patterns relevant to the spread of infectious diseases. PLoS Med. 2008;5(3):e74. doi:10.1371/journal.pmed.0050074 18366252 PMC2270306

[37] Lyngse FP, Mølbak K, Skov RL, ; Danish Covid-19 Genome Consortium. Increased transmissibility of SARS-CoV-2 lineage B.1.1.7 by age and viral load. Nat Commun. 2021;12(1):7251. doi:10.1038/s41467-021-27202-x 34903718 PMC8669007

[38] Lyngse FP, Kirkeby C, Halasa T, . Nationwide study on SARS-CoV-2 transmission within households from lockdown to reopening, Denmark, 27 February 2020 to 1 August 2020. Euro Surveill. 2022;27(6):2001800. doi:10.2807/1560-7917.ES.2022.27.6.2001800 35144726 PMC8832519

[39] Li F, Li YY, Liu MJ, . Household transmission of SARS-CoV-2 and risk factors for susceptibility and infectivity in Wuhan: a retrospective observational study. Lancet Infect Dis. 2021;21(5):617-628. doi:10.1016/S1473-3099(20)30981-6 33476567 PMC7833912

[40] Lyngse FP, Mølbak K, Denwood M, . Effect of vaccination on household transmission of SARS-CoV-2 Delta variant of concern. Nat Commun. 2022;13(1):3764. doi:10.1038/s41467-022-31494-y 35773247 PMC9244879

[41] Lyngse FP, Mortensen LH, Denwood MJ, . Household transmission of the SARS-CoV-2 Omicron variant in Denmark. Nat Commun. 2022;13(1):5573. doi:10.1038/s41467-022-33328-3 36151099 PMC9508106

[42] Lyngse FP, Kirkeby CT, Halasa T, . COVID-19 transmission within danish households: a nationwide study from lockdown to reopening. medRxiv. Preprint posted online September 9, 2020. doi:10.1101/2020.09.09.20191239

[43] Espenhain L, Tribler S, Sværke Jørgensen C, Holm Hansen C, Wolff Sönksen U, Ethelberg S. Prevalence of SARS-CoV-2 antibodies in Denmark: nationwide, population-based seroepidemiological study. Eur J Epidemiol. 2021;36(7):715-725. doi:10.1007/s10654-021-00796-8 34420152 PMC8380416

[44] Rowe BR, Canosa A, Drouffe JM, Mitchell JBA. Simple quantitative assessment of the outdoor versus indoor airborne transmission of viruses and COVID-19. Environ Res. 2021;198:111189. doi:10.1016/j.envres.2021.111189 33872644 PMC8051020

