# Supplementary material for: Children’s Birthday Gatherings and SARS-CoV-2 Infection in Grandparents
Source: JAMA Netw Open. 2026 Jul 13;9(7):e2623042. doi: 10.1001/jamanetworkopen.2026.23042 (PMC13366199; doi:10.1001/jamanetworkopen.2026.23042)
Supplement: Supplement 1. — eTable 1. Hazard ratio of birthday of a grandchild eTable 2. Hazard ratio of birthday of a grandchild by age of the grandchild eTable 3. Hazard ratio of birthday of a grandchild by geographical distance between the grandparent and the grandchild eTable 4. Overall and by variant-period: HR and 95%CI of cox proportional hazard regression of the effect of a grandchild’s birthday eFigure. Flow diagram [file jamanetwopen-e2623042-s001.pdf]

**Supplemental Online Content**

Espenhain L, Mortensen LH, Christiansen LE, Hansen CH, Ethelberg S. Children's birthday gatherings and SARS-CoV-2 infection in grandparents. *JAMA Netw Open*. 2026;9(7):e2623042. doi:10.1001/jamanetworkopen.2026.23042

eTable 1. Hazard ratio of birthday of a grandchild

eTable 2. Hazard ratio of birthday of a grandchild by age of the grandchild

eTable 3. Hazard ratio of birthday of a grandchild by geographical distance between the grandparent and the grandchild

eTable 4. Overall and by variant-period: HR and 95%CI of cox proportional hazard regression of the effect of a grandchild's birthday

eFigure 1. Flow diagram

This supplemental material has been provided by the authors to give readers additional information about their work.

© 2026 Espenhain L et al. *JAMA Network Open*.

**S Table 1 – Hazard ratio of birthday of a grandchild**

Overall and by variant-period: HR and 95%CI of cox proportional hazard regression of the effect of birthday of a grandchild (adjusted for number of grandchildren by stratifying the baseline hazard on the number of grandchildren the grandparent had (1-5)), N grandparents, number of positive grandparents and p-value from Schoenfeld's test for proportional hazards

| Period | HR | CI95% lower | CI95% higher | Number of grandparents | Number of positive grandparents | p-value Schoenfeld's test for proportional hazards |
|---|---|---|---|---|---|---|
| Overall | 1.10 | 1.08 | 1.12 | 1,106,493 | 304,572 | 0.48 |
| Index | 1.07 | 1.00 | 1.13 | 1,098,452 | 28,929 | 0.02 |
| Alpha | 1.04 | 0.93 | 1.16 | 1,019,284 | 8,223 | 0.43 |
| Delta | 1.11 | 1.04 | 1.18 | 1,005,625 | 22,768 | 0.34 |
| Omicron | 1.11 | 1.09 | 1.14 | 971,516 | 228,858 | 0.00 |

© 2026 Espenhain L et al. *JAMA Network Open*.

**S Tabel 2 – Hazard ratio of birthday of a grandchild by** ***age of the grandchild***

Overall and by variant-period: HR and 95%CI of cox proportional hazard regression of the effect of birthday by age of the birthday child (adjusted for which age category (school-aged and non-school aged) the grandparent had grandchildren in and age of the grandparent in four categories and for number of grandchildren by stratifying the baseline hazard on the number of grandchildren the grandparent had (1-5)), N grandparents, number of positive grandparents and p-value from Schoenfeld's test for proportional hazards

| Period | Age of grandchild having birthday | HR | CI95% lower | CI95% higher | p-value Schoenfeld's test for proportional hazards | Number of grandparents | Number of positive grandparents |
|---|---|---|---|---|---|---|---|
| Overall | non school-aged | 1.15 | 1.12 | 1.18 | 0.13 | | |
| | school-aged | 1.07 | 1.04 | 1.09 | 0.46 | 1,106,493 | 304,572 |
| Index | non school-aged | 1.20 | 1.10 | 1.31 | 0.05 | 1,098,452 | 28,929 |
| | school-aged | 0.95 | 0.87 | 1.04 | 0.16 | | |
| Alpha | non school-aged | 1.10 | 0.95 | 1.28 | 0.50 | 1,019,284 | 8,223 |
| | school-aged | 1.01 | 0.85 | 1.20 | 0.69 | | |
| Delta | non school-aged | 1.06 | 0.97 | 1.16 | 0.82 | 1,005,625 | 22,768 |
| | school-aged | 1.14 | 1.05 | 1.25 | 0.17 | | |
| Omicron | non school-aged | 1.16 | 1.13 | 1.20 | 0.01 | 971,516 | 228,858 |
| | school-aged | 1.09 | 1.06 | 1.12 | 0.32 | | |

non school-aged = 1-6 year birthday; school-aged = 7-16 year birthday

© 2026 Espenhain L et al. *JAMA Network Open*.

**S Tabel 3 – Hazard ratio of birthday of a grandchild by geographical distance between the grandparent and the grandchild**

Overall and by variant-period: HR and 95%CI of cox proportional hazard regression of the effect of birthday by distance to the birthday child (adjusted for which distance-category the grandparent had grandchildren in and for number of grandchildren by stratifying the baseline hazard on the number of grandchildren the grandparent had (1-5)), N grandparents, number of positive grandparents and p-value from Schoenfeld's test for proportional hazards

| Period | Distance between grandparent and the birthday child | aHR for birthday | CI95% lower | CI95% higher | p-value Schoenfeld's test for proportional hazards | Number of grandparents | Number of positive grandparents |
|---|---|---|---|---|---|---|---|
| Overall | <25 km | 1.12 | 1.10 | 1.15 | 0.01 | | |
| | 25-149 km | 1.05 | 1.01 | 1.08 | 0.04 | 1,106,493 | 304,572 |
| | ≥150 km | 1.11 | 1.05 | 1.19 | 0.26 | | |
| Index | <25 km | 1.12 | 1.04 | 1.20 | 0.27 | | |
| | 25-149 km | 0.98 | 0.87 | 1.10 | 0.12 | 1,098,452 | 28,929 |
| | ≥150 km | 0.97 | 0.77 | 1.21 | 0.04 | | |
| Alpha | <25 km | 0.99 | 0.86 | 1.14 | 0.16 | | |
| | 25-149 km | 1.15 | 0.94 | 1.41 | 0.55 | 1,019,284 | 8,223 |
| | ≥150 km | 1.03 | 0.67 | 1.57 | 0.78 | | |
| Delta | <25 km | 1.08 | 0.99 | 1.17 | 0.13 | | |
| | 25-149 km | 1.17 | 1.04 | 1.31 | 0.50 | 1,005,625 | 22,768 |
| | ≥150 km | 1.09 | 0.87 | 1.36 | 0.05 | | |
| Omicron | <25 km | 1.14 | 1.11 | 1.17 | 0.00 | | |
| | 25-149 km | 1.06 | 1.02 | 1.10 | 0.69 | 971,516 | 228,858 |
| | ≥150 km | 1.12 | 1.05 | 1.21 | 0.78 | | |

© 2026 Espenhain L et al. *JAMA Network Open*.

**S Tabel 4 i - ix – Overall and by variant-period: HR and 95%CI of cox proportional hazard regression of the effect of a grandchild's birthday** adjusted for number of grandchildren by stratifying the baseline hazard on the number of grandchildren the grandparent had (1-5)), N grandparents, number of positive grandparents and p-value from Schoenfeld's test for proportional hazards:

**i – amongst those age 59 and younger**

| Period | HR | CI_l | CI_h | N gp | zph |
|---|---|---|---|---|---|
| Overall | 1.06 | 1.03 | 1.10 | 287709 | 0.13 |
| Index | 1.06 | 0.95 | 1.18 | 287160 | 0.27 |
| Alpha | 0.98 | 0.81 | 1.18 | 240550 | 0.84 |
| Delta | 0.98 | 0.85 | 1.12 | 224082 | 0.42 |
| Omicron | 1.09 | 1.04 | 1.13 | 198460 | 0.78 |

**ii – amongst those age 60-66**

| Period | HR | CI_l | CI_h | N gp | zph |
|---|---|---|---|---|---|
| Overall | 1.11 | 1.07 | 1.14 | 349167 | 0.42 |
| Index | 1.10 | 0.98 | 1.22 | 313285 | 0.02 |
| Alpha | 1.19 | 0.98 | 1.44 | 266588 | 0.18 |
| Delta | 1.16 | 1.03 | 1.31 | 262797 | 0.85 |
| Omicron | 1.11 | 1.07 | 1.16 | 248207 | 0.07 |

**iii – amongst those age 67-74**

| Period | HR | CI_l | CI_h | N gp | zph |
|---|---|---|---|---|---|
| Overall | 1.14 | 1.10 | 1.18 | 413754 | 0.43 |
| Index | 0.99 | 0.87 | 1.13 | 368602 | 0.52 |
| Alpha | 1.07 | 0.84 | 1.37 | 320903 | 0.90 |
| Delta | 1.10 | 0.98 | 1.24 | 319590 | 0.30 |
| Omicron | 1.17 | 1.12 | 1.21 | 309227 | 0.00 |

**iv – amongst those age 75 years and older**

| Period | HR | CI_l | CI_h | N gp | zph |
|---|---|---|---|---|---|
| Overall | 1.15 | 1.09 | 1.21 | 268355 | 0.30 |
| Index | 1.16 | 0.99 | 1.36 | 225512 | 0.64 |
| Alpha | 0.96 | 0.63 | 1.47 | 206037 | 0.90 |
| Delta | 1.23 | 1.05 | 1.45 | 219407 | 0.85 |
| Omicron | 1.15 | 1.08 | 1.22 | 226467 | 0.06 |

**v – amongst female grandparents**

| Period | HR | CI_l | CI_h | N gp | zph |
|---|---|---|---|---|---|
| Overall | 1.10 | 1.07 | 1.13 | 599721 | 0.91 |
| Index | 1.08 | 0.99 | 1.17 | 595537 | 0.04 |
| Alpha | 0.99 | 0.85 | 1.16 | 553170 | 0.17 |
| Delta | 1.09 | 0.99 | 1.19 | 546574 | 0.70 |
| Omicron | 1.12 | 1.09 | 1.15 | 529073 | 0.01 |

© 2026 Espenhain L et al. *JAMA Network Open*.

**vi – amongst male grandparents**

| Period | HR | CI_l | CI_h | N gp | zph |
|---|---|---|---|---|---|
| Overall | 1.10 | 1.07 | 1.13 | 506772 | 0.33 |
| Index | 1.06 | 0.96 | 1.16 | 502915 | 0.26 |
| Alpha | 1.10 | 0.93 | 1.29 | 466114 | 0.80 |
| Delta | 1.13 | 1.03 | 1.25 | 459051 | 0.34 |
| Omicron | 1.10 | 1.07 | 1.14 | 442443 | 0.02 |

**vii – amongst grandparents with 1 grandchild**

| Period | HR | CI_l | CI_h | N gp | zph |
|---|---|---|---|---|---|
| Overall | 1.09 | 1.03 | 1.14 | 417141 | 0.87 |
| Index | 1.06 | 0.91 | 1.23 | 383930 | 0.21 |
| Alpha | 0.99 | 0.73 | 1.36 | 301357 | 0.05 |
| Delta | 1.21 | 1.02 | 1.43 | 296815 | 0.79 |
| Omicron | 1.08 | 1.02 | 1.15 | 317032 | 0.87 |

**viii – amongst grandparents with 2-4 grandchildren**

| Period | HR | CI_l | CI_h | N gp | zph |
|---|---|---|---|---|---|
| Overall | 1.10 | 1.08 | 1.13 | 733584 | 0.09 |
| Index | 1.14 | 1.05 | 1.23 | 719670 | 0.07 |
| Alpha | 1.03 | 0.90 | 1.18 | 646096 | 0.45 |
| Delta | 1.09 | 1.01 | 1.18 | 637879 | 0.51 |
| Omicron | 1.11 | 1.09 | 1.14 | 626201 | 0.00 |

**ix – amongst grandparents with 5 grandchildren**

| Period | HR | CI_l | CI_h | N gp | zph |
|---|---|---|---|---|---|
| Overall | 1.09 | 1.04 | 1.14 | 99648 | 0.16 |
| Index | 0.92 | 0.81 | 1.05 | 93568 | 0.05 |
| Alpha | 1.14 | 0.86 | 1.50 | 71831 | 0.17 |
| Delta | 1.09 | 0.92 | 1.30 | 70931 | 0.39 |
| Omicron | 1.13 | 1.07 | 1.19 | 74669 | 0.20 |

© 2026 Espenhain L et al. *JAMA Network Open*.

**S Figure 1: Flow diagram**

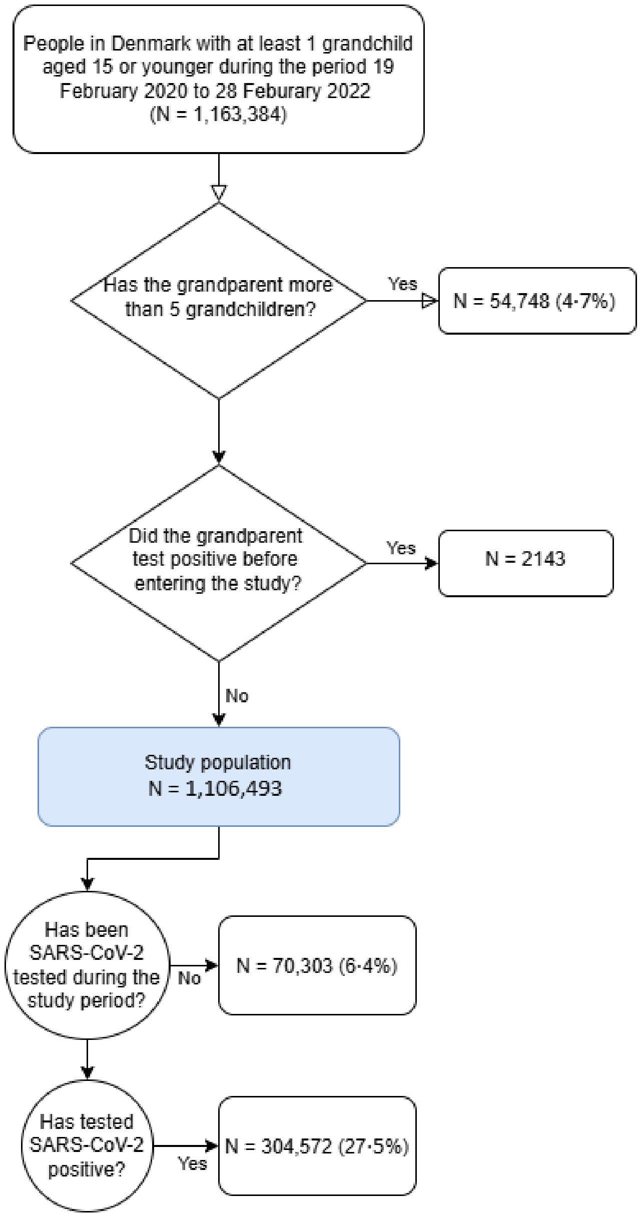


© 2026 Espenhain L et al. *JAMA Network Open*.
