# Supplementary material for: Children’s Birthday Gatherings and SARS-CoV-2 Infection in Grandparents
Source: JAMA Netw Open. 2026 Jul 13;9(7):e2623042. doi: 10.1001/jamanetworkopen.2026.23042 (PMC13366199; doi:10.1001/jamanetworkopen.2026.23042)
Supplement: Supplement 2. — Data Sharing Statement [file jamanetwopen-e2623042-s002.pdf]

Data Sharing Statement

Espenhain. Children's Birthday Gatherings and SARS-CoV-2 Infection in Grandparents. *JAMA Netw Open*. Published July 13, 2026. doi:10.1001/jamanetworkopen.2026.23042

Data
Data available: Yes
Data types: Participant data with identifiers
How to access data: The data utilized in this study is accessible under restricted conditions under Danish data protection laws. No data collection was conducted specifically for this study. The data are available for access to members of the scientific community complying with Danish data protection regulations with any necessary permissions and for non-commercial use only, upon reasonable request to the authors and with permission of Statistics Denmark (DST).
When available: With publication

Supporting Documents
Document types: None

Additional Information
Who can access the data: The data utilized in this study is accessible under restricted conditions under Danish data protection laws. No data collection was conducted specifically for this study. The data are available for access to members of the scientific community complying with Danish data protection regulations with any necessary permissions and for non-commercial use only, upon reasonable request to the authors and with permission of Statistics Denmark (DST).
Types of analyses: The data utilized in this study is accessible under restricted conditions under Danish data protection laws. No data collection was conducted specifically for this study. The data are available for access to members of the scientific community complying with Danish data protection regulations with any necessary permissions and for non-commercial use only, upon reasonable request to the authors and with permission of Statistics Denmark (DST).
Mechanisms of data availability: The data utilized in this study is accessible under restricted conditions under Danish data protection laws. No data collection was conducted specifically for this study. The data are available for access to members of the scientific community complying with Danish data protection regulations with any necessary permissions and for non-commercial use only, upon reasonable request to the authors and with permission of Statistics Denmark (DST).
